# Incipient need of targeting airway remodeling using advanced drug delivery in chronic respiratory diseases

**DOI:** 10.4155/fmc-2020-0091

**Published:** 2020-04-30

**Authors:** Meenu Mehta, Saurabh Satija, Keshav R Paudel, Gang Liu, Dinesh K Chellappan, Philip M Hansbro, Kamal Dua

**Affiliations:** ^1^Discipline of Pharmacy, Graduate School of Health, University of Technology Sydney, NSW 2007, Australia; ^2^Center for Inflammation, Centenary Institute, Sydney, NSW 2050, Australia; ^3^School of Pharmaceutical Sciences, Lovely Professional University, Phagwara 144411, Punjab, India; ^4^School of Life Sciences, University of Technology Sydney, Ultimo, NSW 2007, Australia; ^5^Department of Life Sciences, School of Pharmacy, International Medical University, Bukit Jalil 57000, Kuala Lumpur, Malaysia; ^6^Priority Research Centre for Healthy Lungs, University of Newcastle & Hunter Medical Research Institute, Lot 1 Kookaburra Circuit, New Lambton Heights, Newcastle, NSW 2305, Australia; ^7^School of Pharmaceutical Sciences, Shoolini University, Solan, Himachal Pradesh 173229, India

**Keywords:** COPD, lung cancer, remodeling, respiratory disorders

Advancements in the field of drug delivery, particularly nanoparticles, is providing an extra edge in combating the emerging complications of airway remodeling in chronic respiratory diseases.

Several million people around the globe are affected by chronic respiratory disorders (CRDs) such as asthma, chronic obstructive pulmonary disease (COPD), TB and lung cancer. This also includes, around 500 million people from developed countries who suffer from these CRDs [1]. CRDs are typically diseases related to lung airways. The prevalence of CRDs in children and especially in the elderly, is rapidly growing and the associated strains have negatively affected many people’s lives [2]. The WHO data suggests that, the mortality rate due to CRDs was 4.6 million in the past and has reported concerns that this number might rise dramatically in the future. Few of the important triggering factors involved in these CRDs include allergens, air pollution and smoking tobacco [3]. Moreover, global pandemics such as the coronavirus disease (COVID-19) may also contribute to exacerbate the conditions of various CRDs, particularly by triggering the airway remodeling features.

## Airway remodeling

Chronic respiratory diseases primarily occur due to radical changes in the respiratory tract called as remodeling [4]. In the pathophysiology of the respiratory system, remodeling is specifically concerned with the occurrence of highly composite structural transformations that affect the airways, such as, disruption of the epithelial cells, inflammatory cell infiltration and apparent thickening of the basement membrane due to collagen deposition resulting in increased mucus glands secretions [5]. This process of continued disruption and modification of structural cells and tissues leading to the development of a new airway-wall, and as a result, to an altered physiology, is known as airway remodeling [6]. These mechanisms are of much interest in terms of the pathogenesis of asthma and are primarily influenced by principles of immunology and inflammation in the debate regarding asthma causation. While there are well-known airway structural changes in chronic COPD, much less attention is paid to the pathology of the disease. This is likely because they are more superficial and they overshadow the neighbouring emphysematous tissue destruction [7].

There are several ‘remodeling’ processes; however, the changes found in proportion are very different. Such modifications include fibrosis of the airways, decreased smooth muscle mass, mucous metaplasia, hypertrophy of the glandulas, as well as, lesser well-defined changes in bronchial vasculature and nerves. In the case of asthma, bronchial portions and subsegmental walls have their whole dimensions thickened [8]. In COPD, only the inner wall of the major airways is thicker and more persuasive. The peripheral airways are often distinctly remodeled in COPD, typically free of cartilage or bronchial tissue [9,10].

## Advanced drug delivery for regeneration

Like most airway remodeling, transient airflow obstructions, caused by inflammation, mucus spikes and bronchial hyper-reactiveness are associated with CRDs, for instance. Given the present care and management choices, a substantial number of patients remain poorly managed for such diseases, namely, asthma and COPD, which are usually triggered by a respiratory virus infection. As a result, new innovative drug therapies remain important in order that exacerbations can be better managed and avoided. Thus, various advanced therapies have been developed in the field of drug delivery in respiratory diseases such as nanoparticles (NPs), including extracellular vesicles and their synthetic equivalents [11].

Ideally, these new therapeutic strategies are focused on the activation of the regenerative capacity of the lung itself. Understanding the various pathways and the targeted delivery of drugs that initiate, sustain, modulate, and conclude normal lung development could be essential to new regenerative approaches by reactivating pulmonary disease pathways [12].

Targeted advanced drug-delivery strategies can provide increased accumulation, greater effectiveness and enhanced protection. Gabriela *et al.* recently coupled an anti-fibrotic small molecule (αPV1) with an anti-PV1 antibody and reported a substantial reduction in lung fibrosis in idiopathic pulmonary fibrosis, compared with an isotype controlled antibody [13]. Juan *et al.* made *ATG101* single-stranded antisense RNA-loaded DNA triangular NPs (ss*ATG101*-TNP) to knock down expression of the *ATG101* gene. They demonstrated that ss*ATG101*-TNP can efficiently be transfected into human pulmonary arterial endothelial cells in a time and dose-dependent manner, and knockdown of *ATG101* stimulates the cell apoptosis and inhibits hypoxic cell autophagy and proliferation as a possible therapeutic goal for endothelial injury related conditions [14]. In scleroderma associated interstitial lung disease (SSc-ILD), patients with derived cells in an experimental lung fibrosis model, were administered with imatinib loaded gold nanoparticles (GNPs). GNPs were synthesized using anti-CD44 and were loaded with imatinib (GNP–HCIm). Patients with scleroderma associated interstitial lung disease were diagnosed with lung fibroblast and alveolar macrophages in the presence of NPs from bronchoalveolar lavage fluids. Their research showed that the GNP–HCIm significantly inhibited proliferation and viability inducing apoptosis of LFs and effectively reduced IL-8 release, viability and M2 polarization in alveolar macrophages. [15].

Tsai *et al.* reported that cerium dioxide NPs can reduce Ca^2+^ cytosolic change and TiO_2_ NP-induced mitochondrial damage. Their team presented evidence that TiO_2_ NPs can attenuate hypersecretion and apoptosis progression [16]. In a most recent study conducted by Chattopadhyay *et al.*, atropine nanoparticles (ANPs) have been shown to suppress inflammatory cytokines, reduce shallow breathing and normalize the hyper-responsiveness of the tidal tissue and obstructed lungs. Moreover, treatment with ANP reduced progressive blockage of the airway and decreased deposition of collagens. Thus, ANP strengthens the airway surfaces of the lung and reduces lung hyperaction, blockage and inflammation [17]. Lou *et al.* showed the significance of miRNA in airway remodeling. It was shown that miR-192-5p had an overexpressed effect in the smooth muscle cells in airways. In addition, *in vitro* and *in vivo* asthma mice demonstrated a similar effect which was shown to be the effect of miR-192-5p on proliferation [18]. Prior to this, various other studies have also reported the potential of miRNA in tissue regeneration. Simeoli *et al.* in their research showed that the delivery of LNA-based anti-miR21 and anti-miR-712 in mouse models of atherosclerosis and nerve trauma reduced the inflammatory macrophage number through liposomes or cationic lipids-coated NPs [19,20]. All of these evidences in one way or other, state that the combinations of the different targeted approach contribute to the drug research and development for airway tissue regeneration.

## Conclusion

Advancements in the field of drug delivery, particularly NPs, is providing an extra edge in combating the emerging complications of airway remodeling in chronic respiratory diseases. Considering the fact that remodeling worsens the respiratory disease pathology, it is an emerging and demanding area of research to be explored by translational, clinical and drug-delivery scientists to provide a new direction to the pulmonary clinics especially during the current, complex and uncertain times of global pandemic situation.
